# An intersectionality perspective on tuberculosis: social determinants affecting tuberculosis mortality rate in Ecuador

**DOI:** 10.3389/fpubh.2025.1659887

**Published:** 2025-10-24

**Authors:** Ángel Sebastián Rodríguez-Pazmiño, Darwin Paredes-Núñez, Daniel Ramos-Sarmiento, Karina Lalangui-Vivanco, Solon Alberto Orlando, Alexandra Narvaez, Greta Franco-Sotomayor, Miguel Angel Garcia-Bereguiain

**Affiliations:** ^1^One Health Research Group, Universidad de Las Américas, Quito, Ecuador; ^2^Centro de Investigación en Salud Pública y Epidemiología Clínica (CISPEC), Facultad de Ciencias de la Salud Eugenio Espejo, Universidad UTE, Quito, Ecuador; ^3^Facultad de Medicina Veterinaria y Agronomía, Universidad UTE, Santo Domingo, Ecuador; ^4^Instituto Nacional de Salud Pública e Investigación, Guayaquil, Ecuador; ^5^Universidad Ecotec, Guayaquil, Ecuador; ^6^Universidad Espíritu Santo, Guayaquil, Ecuador; ^7^Universidad Católica de Santiago de Guayaquil, Guayaquil, Ecuador

**Keywords:** tuberculosis, Ecuador, social determinants, poverty, rurality, parishes

## Abstract

**Introduction:**

The Intersectionality approach to studying tuberculosis (TB) is a complex one. While historical data and indicators consistently suggest a positive correlation between social determinants, such as poverty, and TB mortality, the strength of this association varies across different regions and countries. Overcrowding and population density are also recognized as risk factors for TB transmission.

**Methods:**

In this study, we conducted a descriptive and observational statistical analysis of TB mortality in Ecuador using the most recent public data from 2010. We examined the association between TB mortality and poverty, as well as territorial distribution, population density, and overcrowding.

**Results:**

Our univariate analysis results indicate that the rural parishes or canton capitals in the first quintile of poverty (Q1) had the highest average mortality rates (14.23 per 100,000 inhabitants). Furthermore, the average TB mortality ratio was substantially higher in rural areas compared to urban ones (12.72 vs. 7.5 per 100,000 inhabitants). Interestingly, zones with the highest population density had a significantly lower average TB mortality ratio than those with the lowest density (4.82 vs. 15.19 per 100,000 inhabitants). Likewise, overcrowding analysis reveals a significant difference between the group with the highest level (O1) vs. the lowest one (O5; 14.3 vs. 6.8 per 100,000 inhabitants). On the other hand, a multivariate linear regression model agrees that three of the four independent variables evaluated had statistically significant associations with tuberculosis mortality rate. The percentage of poverty, living in a rural area, and population density were significant predictors of higher mortality. In contrast, the level of overcrowding, as determined by multivariate analysis, did not show a significant association when the other independent variables were taken into account.

**Discussion:**

These findings reinforce the strong link between TB mortality and poverty, rurality, a discreet relationship with overcrowding, and an inverse relationship with population density in the Ecuadorian context, highlighting the need for targeted public health interventions in rural underserved communities. Future research should explore how changes in socioeconomic conditions and healthcare access have influenced TB incidence.

## 1 Introduction

Tuberculosis (TB), a disease caused by the *Mycobacterium tuberculosis* complex (MTBC), has very ancient origins (1) and persists as a significant public health threat in much of the world, especially in low- and middle-income countries (LMICs) (2). The World Health Organization (WHO) estimated that 10.8 million people were infected with TB worldwide in 2023. This was the highest number of cases recorded since the WHO began monitoring TB in 1995 (3). In Ecuador, the TB incidence rate in 2023 was around 58 cases per 100,000 population, indicating a worrying 47% increase over 2015 (4), the year in which the WHO started the End TB Strategy.

One of the pillars of the WHO's “End TB Strategy” states that it is critical to study TB from an intersectionality perspective, which means to address the social determinants of TB among vulnerable groups, such as people living in poverty (5). Particularly, extreme poverty has been evidenced as a rural phenomenon (6). In 2013, it was estimated that four out of every five people living on <$1.90 per day inhabited rural communities (7). On the other hand, high population density and high levels of household overcrowding are factors that can facilitate the spread of TB (8–10).

Intersectionality, where often underestimated factors reinforce each other, identifies poverty as a key social determinant of health, particularly TB, influenced by limited access to medical services, food insecurity, chronic malnutrition, and poor housing conditions (11–13). Lönnroth et al. demonstrated that overcrowding caused by poverty increases exposure to *Mycobacterium tuberculosis* Complex and weakens the immune response, favoring the progression from latent to active TB. They also concluded that intersectoral interventions aimed at improving socioeconomic conditions can significantly reduce the TB burden (14). Similarly, in India, TB mortality rates were found to be considerably higher in communities with low income and restricted access to health services (15). In Latin American contexts, research by Munayco et al. (16) showed that the combination of poverty, rurality, and structural barriers (such as geographic distance to health centers, shortage of trained personnel, and intermittent availability of medicines) is associated with delays in diagnosis, treatment interruptions, and higher TB mortality.

In Ecuador, poverty is quantified by various criteria through the “Instituto Nacional de Estadísticas y Censos” (INEC). One of them, poverty by unsatisfied basic needs (UBN), is measured based on economic capacity, access to basic education, housing, basic services, and overcrowding (17). Poverty by UBN is determined at the national, provincial, cantonal, and parish levels. INEC has also presented research on population density and overcrowding levels up to the parish level in 2010 and 2022, years in which national censuses were conducted in Ecuador (18, 19).

Nevertheless, a gap remains in national and international literature regarding the integrated analysis of these determinants under an intersectional approach and at high-resolution subnational scales. The absence of studies that simultaneously consider the interaction between social, geographic, and structural factors limits the understanding of TB mortality distribution patterns and the identification of populations at extreme risk.

In this context, the present research poses the central question: how do poverty, rurality, population density, and overcrowding influence TB mortality at the parish and cantonal capital level in Ecuador? Consequently, the study aims to analyze the relationships between TB mortality and specific social determinants, including poverty (measured through Unsatisfied Basic Needs, UBN), geographic area (urban or rural), population density, and overcrowding, using parish and cantonal capital level data from Ecuador for the year 2010 (most up-to-date information for the purposes of this study).

## 2 Methodology

### 2.1 Study design

This study is an observational ecological study in which we analyzed disaggregated data from 2010 at the parish and cantonal capital level. Ecuador's territorial division consists of provinces, cantons, and parishes. We utilized data from the INEC on poverty, measured by UBN, and territorial classification (urban or rural) based on the country's political-administrative division, as defined by the “Dirección de Cartografía Estadística y Operaciones de Campo” (20). Regarding territorial classification, INEC adds urban parishes in cantonal capitals to its databases. Rural parishes are treated as separate territorial units. This is why the comparative analyses in this study are carried out between rural parishes and cantonal capitals (urban areas). Additionally, we included population density and overcrowding data from the “Censo de Población y Vivienda” (21).

Poverty, based on UBN, a variable that aggregates multiple socioeconomic indicators, is represented as a percentage, with higher figures indicating greater poverty. In terms of territorial division, we have the categories “urban” and “rural.” Population density is expressed as the number of inhabitants per square kilometer. Finally, overcrowding refers to the condition in which the number of individuals residing in a dwelling exceeds the adequate capacity of habitable space, potentially increasing the risk of transmission of infectious diseases such as TB. According to this, the INEC measures overcrowding as the percentage of households in which the number of occupants per room designated for sleeping surpasses a defined threshold, generally considered to be more than two persons per room (18, 19).

These social determinant variables were compared with the rural parishes and cantonal level TB mortality cases data obtained from the “Registro Estadístico de Defunciones Generales” (22), published by the INEC. This database compiles information from death certificates issued by the Ecuadorian Civil Registry. Subsequently, TB mortality ratios were calculated as the number of deaths divided by the parish population, multiplied by 100,000. Upper and lower 95% confidence intervals were also considered for the TB mortality ratio and recorded in Supplementary Material 1.

### 2.2 Descriptive analysis

A matrix was constructed integrating the variables considered in this study (see Supplementary Material 1), including:

- Zone (rural parishes or canton capitals) codes and names (columns A and B).
- Rural parish or canton capital population, number of TB-related deaths, and TB mortality ratio (columns C, D, and E).
- Confidence intervals inferior and superior at 95% of the TB mortality ratio (columns F and G).
- Territorial division as urban or rural (column H).
- Percentage of poverty by UBN with its classification into five quintiles (Q1–Q5; columns I and J).
- Population density (inhabitants/km^2^) with its corresponding classification into five groups (PD1–PD5 columns K and L).
- Overcrowding levels, also categorized into groups (O1–O5; columns M and N), and
- The provinces corresponding to the parishes and their geographic regions (columns O and P).

From a total of 1,021 parishes (rural zones) and cantonal capitals (urban zones) in Ecuador, 168 zones with recorded TB-related deaths were initially selected. To ensure data quality, zones with missing information for any variable were excluded, resulting in a final dataset of 166 parishes with complete data. Given that 83.7% of the country's zones reported no TB-related deaths during the study period (and thus presented a high concentration of zero values), it was methodologically appropriate to focus the analysis on these 166 parishes. This approach enables a clearer examination of the social and demographic conditions associated with TB mortality, thereby avoiding the dilution of patterns in areas where the phenomenon is absent. It also aligns with analytical strategies that prioritize observed cases and reduce potential biases introduced by zero-inflated data. All relevant information is provided in Supplementary Material 1.

### 2.3 Outliers' assessment

The interquartile range (IQR) criterion was used to identify and eliminate outliers concerning TB mortality ratios. Here, the quartile 1 (25th percentile) and quartile 3 (75th percentile) were calculated, as well as the interquartile range IQR = quartile 3—quartile 1. Values below “quartile 1”−1.5 ^*^ IQR or above “quartile 3” + 1.5 ^*^ IQR were then considered outliers, and these parishes were excluded from subsequent analyses.

### 2.4 Determination of study groups

**Figure 1:**
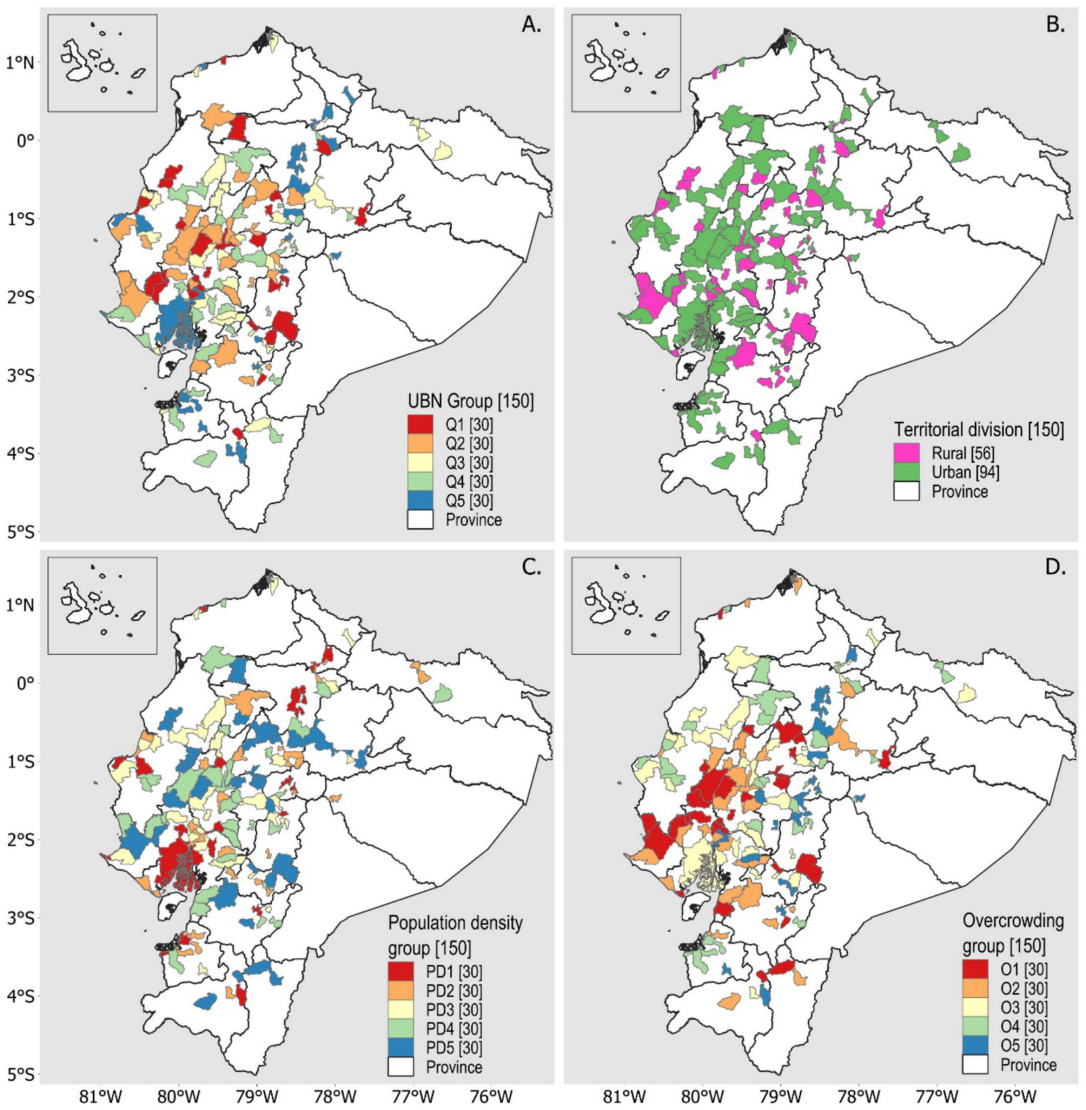
Geographic location of study groups with parishes with cases of TB deaths. **(A)** Distribution of parishes by poverty quintiles according to UBN. **(B)** Distribution of parishes according to the territorial division of urban and rural areas. **(C)** Distribution of parishes according to population density levels. **(D)** Distribution of parishes according to overcrowding levels.

After discarding parishes with outliers, five groups were created at equal intervals for poverty by UBN (Q1 to Q5), five groups for population density (PD1 to PD5), and five groups for levels of overcrowding (O1 to O5). The first group in each category corresponded to the group with the highest values, while group five corresponded to those with the lowest values. The territorial division study groups consisted of the rural parishes (“rural”) or canton capital (“urban”) that make up each zone. The geographic location of the study groups is shown in the maps in Figure 1.

### 2.5 Univariate statistical tests

Four statistical analyses were conducted to compare the results between:

- TB mortality ratios concerning poverty by UBN
- TB mortality ratios concerning the territorial division
- TB mortality ratios concerning population density, and
- TB mortality ratios concerning overcrowding.

The Shapiro-Wilk test was used to assess the normal distribution of the data in all four cases (*p*-value <0.05). Consequently, the nonparametric Kruskal-Wallis test was applied to determine whether at least one study group differed significantly from the others (*p*-value <0.05). Dunn's *post hoc* test was then used to identify specific group differences (*p*-value <0.05). Finally, box plots were generated to visualize the dispersion of the data.

These analyses were performed in R software (v 4.4.3).

### 2.6 Multivariate statistical analysis

To reinforce the identification of factors associated with TB mortality rates, a multiple linear regression model was constructed using the TB mortality ratio as the dependent variable. The analysis was restricted to the zones that reported at least one TB-related death in 2010 (excluding zones with outliers), to avoid distortion caused by a large number of observations with zero mortality (zero-inflated data, 83.7% of all zones of Ecuador), which could dilute real associations between the variables of interest. The independent variables included in the model were: percentage of poverty based on UBN, territorial division (urban or rural), population density (inhabitants per square kilometer), and the percentage of overcrowding. The variable “territorial division” was coded as a categorical variable with two levels: urban and rural. The other three independent variables (poverty, population density, and overcrowding) were categorized into five groups, corresponding to each level.

Due to the skewed distribution of the dependent variable and to improve compliance with linear model assumptions, a natural logarithmic transformation was applied to the mortality rate: log (rate + 1). The initial model was constructed, and assumptions were evaluated using the Breusch-Pagan test for heteroscedasticity and the Shapiro-Wilk test for normality of residuals, complemented by visual inspection of the Q-Q plot.

## 3 Results

### 3.1 Geographical distribution of TB deaths

Ecuador has 24 provinces, 221 cantons, and 1,021 rural parishes and canton capitals (considering the aggregation of urban parishes in cantonal capitals according to INEC data), of which 168 had cases of death from TB in 2010. We eliminated two zones for presenting incomplete information in the variables of our study, resulting in a preliminary group of 166. After applying the interquartile criterion to eliminate zones with outliers in mortality ratios, we obtained a final group of 150 zones.

In that year, a total of 528 TB-related deaths were recorded across the 150 zones. Guayaquil was the zone with the highest number of TB-related deaths (176 cases), followed by Quito with 32 and Milagro with 16. Eighty-nine zones (59.3%) reported only one case of TB mortality. Considering the TB mortality ratio per 100,000 inhabitants, zones in the provinces of Zamora Chinchipe, Guayas, Cañar, Azuay, Cotopaxi, Los Ríos, Esmeraldas, and Manabí showed the highest rates, ranging from 22.22 to 31.72 (top 10 positions: six rural and four urban).

**Figure 2:**
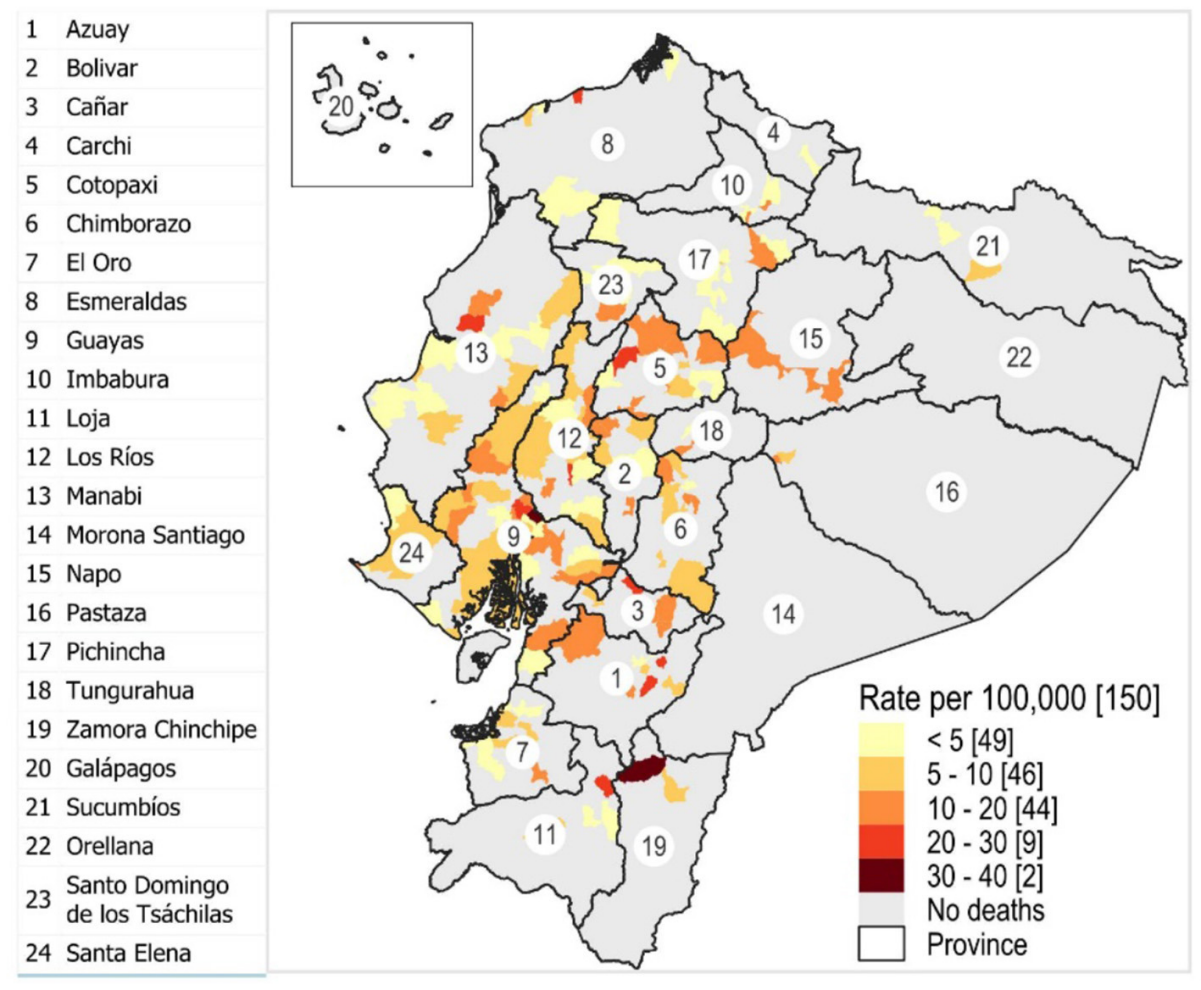
Geographic location of the parishes concerning the TB mortality rate per 100,000 population. The numbers represent the provinces of the country.

At the regional level, 66 zones in the Andean region hosted TB deaths, 75 on the Coast, and nine on the Amazon. At the provincial level, Guayas had 25 zones, Manabí 16, Los Ríos 15, Cotopaxi 11, Pichincha 11, Azuay 7, El Oro 7, Tungurahua 7, Chimborazo 9, Bolívar 6, Cañar 6, Esmeraldas 5, Santa Elena 5, Loja 4, Imbabura 4, Napo 3, Pastaza 2, Santo Domingo De Los Tsáchilas 2, Sucumbíos 2, Zamora Chinchipe 2, and Carchi 1. The TB mortality ratios for the 150 zones, along with their geographic locations, are shown in Figure 2.

### 3.2 Univariate statistical analysis

For the statistical analysis (univariate and multivariate), after excluding the 16 zones with outliers using the interquartile range criterion, a final group of 150 zones remained. This meant that each of the five groups of poverty, population density, and overcrowding had 30 zones. In territorial division, 56 rural parishes and 94 urban zones (aggregated in the cantonal capitals) were identified. The total number of TB deaths for the final group was 528.

#### 3.2.1 Distribution of TB deaths across poverty UBN quintiles

**Figure 3:**
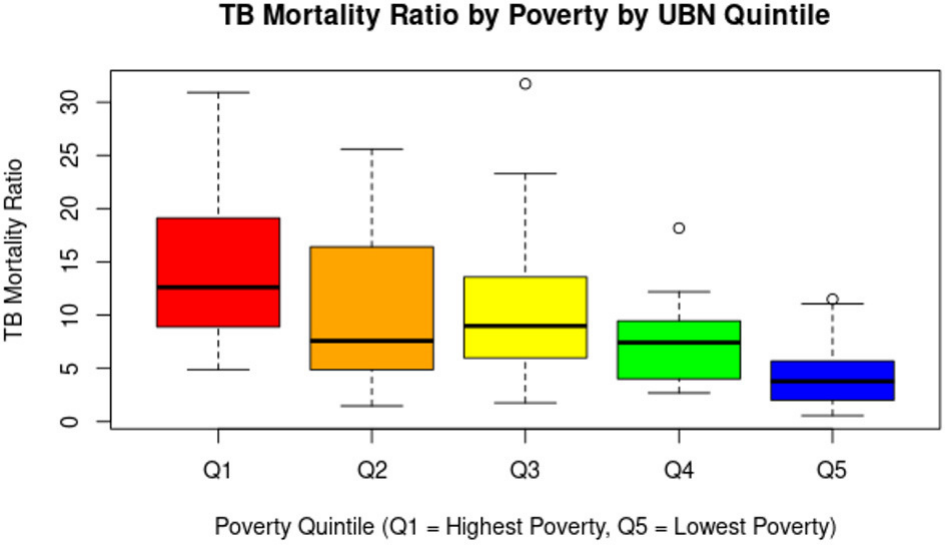
The graph shows a clear downward trend in the tuberculosis mortality rate as poverty levels decrease. Parishes in the poorest quintile (Q1) have the highest median rates and greater dispersion, indicating significant health inequalities associated with socioeconomic status. In contrast, parishes in the least poor quintile (Q5) have considerably lower median rates and less variability, although some outliers are also identified.

TB deaths were distributed unevenly across poverty quintiles, with the highest number occurring in Q5 (less poverty level group, 54.36%, *n* = 287), followed by Q4 (19.51%, *n* = 103), Q3 (10.80%, *n* = 56), Q2 (8.71%, *n* = 46), and Q1 (6.63%, *n* = 35). However, regarding the average TB mortality ratio (data normalized by population), Q1 (the poorest level group) had the highest value at 14.23, while Q2 was 10.76, Q3 was 10.81, Q4 was 7.13, and Q5 was 4.31. The results of the Kruskal–Wallis test, followed by Dunn's *post-hoc* test with Bonferroni correction (*p* < 0.05), show that the poorest group (Q1) has significantly higher TB mortality than the higher socioeconomic groups (Q4 and Q5). These data are detailed in Table 1. A boxplot illustrating these differences is shown in Figure 3.

**Table 1:** Zones (rural parishes or canton capitals) with TB death cases concerning poverty by UBN quintiles.

**Quintil group**	**Number of zones**	**Average poverty (%)**	**TB death cases**	**Deaths (%)**	**Average TB mortality ratio (×100,000)**
Q1	30	95.91	35	6.63	14.23
Q2	30	88.99	46	8.71	10.76
Q3	30	78.53	57	10.80	10.81
Q4	30	66.62	103	19.51	7.13
Q5	30	44.22	287	54.36	4.31
Total	150	–	528	100.00	–

#### 3.2.2 Distribution of TB deaths across urban and rural zones

**Figure 4:**
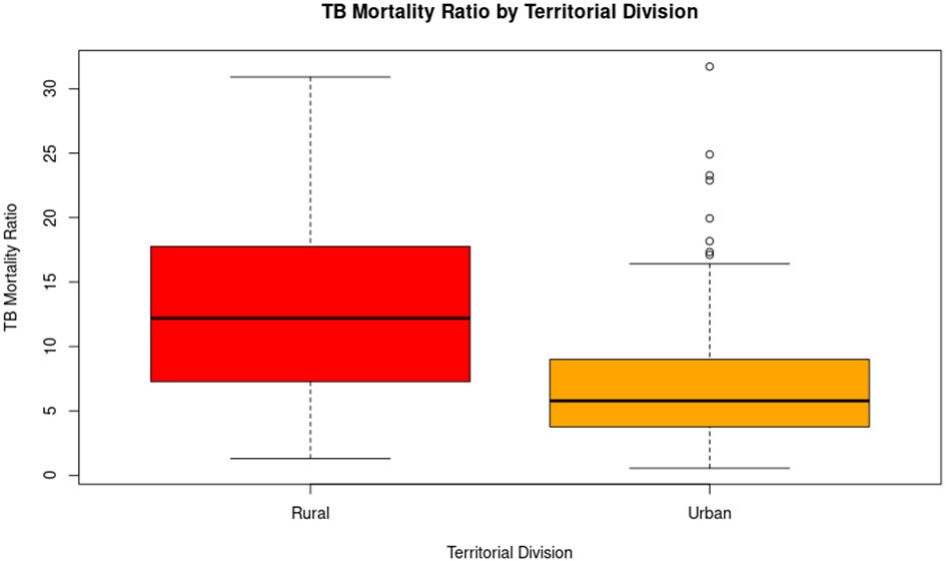
The distribution of tuberculosis mortality rates differs according to territorial division. Rural parishes have a significantly higher median mortality rate than urban parishes, as well as greater overall variability. In contrast, although the median in urban parishes is lower, several outliers are observed, indicating that some specific urban areas also face a disproportionate burden of TB mortality. These results suggest that the rural environment is more affected overall, but that there are urban hotspots that require specific attention.

Among the 150 zones, 94 (62.67%) were urban and 56 (37.33%) were rural. A total of 465 TB-related deaths occurred in urban zones, while 63 were recorded in rural zones. However, the average TB mortality ratio was significantly higher in rural zones (12.72) compared to urban ones (7.5). This disparity is statistically significant (*p* < 0.05) and is further illustrated in Table 2. A boxplot visualizing this difference is presented in Figure 4.

**Table 2:** Parishes with TB death cases concerning territorial division.

**Territorial division**	**Number of parishes**	**Average poverty (%)**	**TB death cases**	**Deaths (%)**	**Average TB mortality ratio (×100,000)**
Urban	94	68.45	465	88.07	7.5
Rural	56	85.6	63	11.93	12.72
Total	150	–	546	100.00	–

#### 3.2.3 Distribution of TB mortality cases by population density

**Figure 5:**
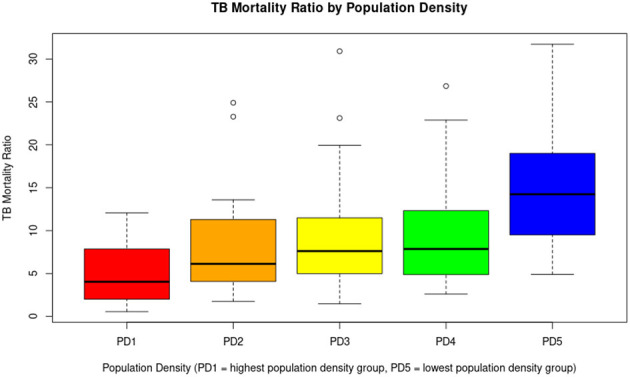
A general inverse trend is observed. As population density decreases (from PD1 to PD5), the median TB mortality tends to increase. Less densely populated parishes (PD5) may be associated with higher tuberculosis mortality, possibly due to limited access to health services, late diagnosis, or unfavorable socioeconomic conditions. Although densely populated areas (PD1) are usually associated with a higher risk of transmission, in this case, they have lower mortality rates, which could be due to better health infrastructure or greater responsiveness of the health system.

The number of TB-related deaths varied across population density groups, with PD1 recording the highest count (325 deaths), followed by PD2 (64), PD3 (61), PD4 (43), and PD5 (35). PD1 represents the group with the highest population density, while PD5 has the lowest. The average TB mortality ratio increased as population density decreased, with values of 4.82 in PD1, 7.82 in PD2, 9.72 in PD3, 9.68 in PD4, and 15.19 in PD5. As population density decreases (from PD1 to PD5), the tuberculosis mortality rate increases significantly according to statistical tests. PD5 (the least densely populated zones) has significantly higher TB mortality rates than all other groups. PD1 (the most densely populated zones) has significantly lower rates compared to PD3, PD4, and PD5. Data are detailed in Table 3, and a boxplot illustrating the differences is presented in Figure 5.

**Table 3:** Parishes with TB death cases concerning population density (PD).

**Population density group**	**Number of parishes**	**Average poverty (%)**	**Average density (inhabitants/km^2^)**	**TB death cases**	**Deaths (%)**	**Average TB mortality ratio (×100,000)**
PD1	30	51.82	1,373.44	325	61.55	4.82
PD2	30	68.29	244.94	64	12.12	7.82
PD3	30	80.63	109.18	61	11.55	9.72
PD4	30	84.01	61.63	43	8.14	9.68
PD5	30	89.52	26.85	35	6.63	15.19
Total	150	–	–	528	100.00	–

#### 3.2.4 Distribution of TB death cases by overcrowding level

**Figure 6:**
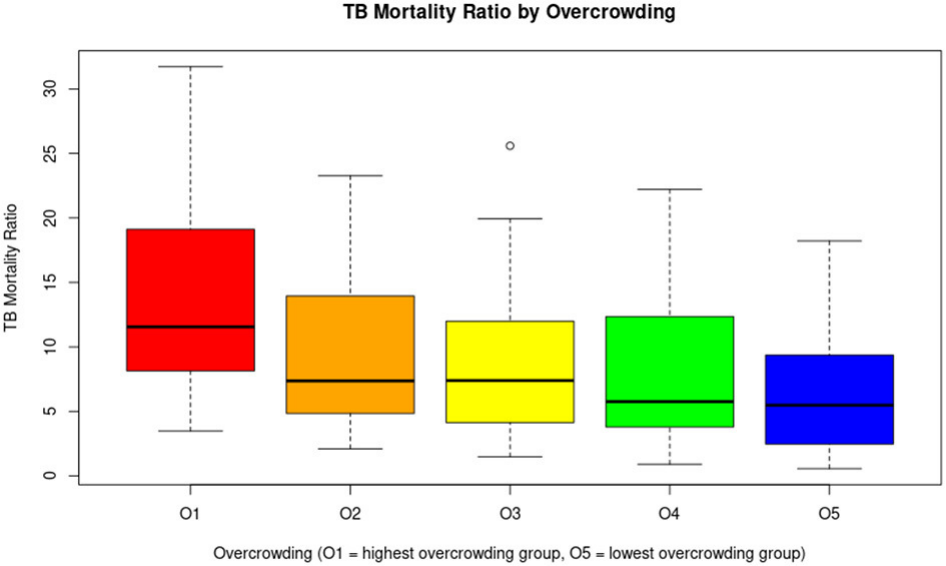
The graph suggests an inverse association between overcrowding and TB mortality rates: areas with higher levels of overcrowding have higher tuberculosis mortality rates. This observation supports the hypothesis that overcrowding is an important social determinant of health in relation to TB.

In this comparison, the number of TB-related deaths varied across overcrowding levels, with O1 reporting 51 deaths, O2 with 61, O3 with 261, O4 with 72, and O5 with 83. O1 represents the group with the highest level of overcrowding, while O5 has the lowest. The average TB mortality ratios were 14.3 in O1, 9.64 in O2, 8.35 in O3, 8.15 in O4, and 6.8 in O5. Statistical analyses revealed a clear statistical difference between O1 and O5 (*p* = 0.0001). These data are detailed in Table 4, and a boxplot illustrating the differences is presented in Figure 6.

**Table 4:** Parishes with TB death cases concerning overcrowding (O).

**Overcrowding group**	**Number of parishes**	**Average poverty (%)**	**Average overcrowding (%)**	**TB death cases**	**Deaths (%)**	**Average TB mortality ratio (×100,000)**
O1	30	91.48	33.11	51	11.36	14.3
O2	30	79.96	25.49	61	12.88	9.64
O3	30	75.73	20.27	261	48.48	8.35
O4	30	73.8	16.04	72	14.20	8.15
O5	30	53.3	10.27	83	16.48	6.8
Total	150	–	–	528	100.00	–

### 3.3 Multivariate analysis results

The results show that poverty was positively and significantly associated with TB mortality (β = 0.011, SE = 0.004, *t* = 2.98, *p* = 0.003), indicating that the higher the proportion of households with poverty by UBN in a zone, the higher the mortality rate. Likewise, rural areas had significantly higher mortality rates than urban areas (β = 0.281, SE = 0.099, *t* = 2.83, *p* = 0.005). In contrast, population density was negatively and significantly associated with mortality (β = −0.00015, SE = 0.00007, *t* = −2.05, *p* = 0.042), suggesting that in more densely populated areas, rates tend to be lower. Overcrowding, although it showed a positive relationship, did not reach statistical significance once the other factors were controlled (β = 0.009, SE = 0.007, *t* = 1.05, *p* = 0.298).

The model explains ~38.4% of the variability in TB mortality (*R*^2^ = 0.384) and was statistically significant as a whole (*F* = 22.59; *p* < 0.001), supporting its usefulness in describing the observed associations.

## 4 Discussion

TB is recognized as a growing public health concern in Ecuador, with an incidence rate of 34.53 per 100,000 inhabitants in 2018, 45 per 100,000 in 2022 (post-COVID-19 pandemic), and 58 per 100,000 in 2023 (23, 24). Similarly, an increasing trend is observed in other countries of the region (23, 25). A clear example is Peru, which recorded the highest incidence in South America in 2023, with 173 cases per 100,000 inhabitants, reflecting a steady increase from 151 per 100,000 in 2022 and 119 per 100,000 in 2018 (23, 26). This regional trend of growing TB incidence has been associated with the prolonged duration of treatment and inadequate healthcare supervision, leading to high dropout treatment rates (21, 22). Moreover, reduced access to medical services hinders timely diagnosis and treatment, particularly in rural and underserved urban areas (27–30). Studies in Latin America have shown a correlation between the percentage of GDP allocated to healthcare and the incidence of TB, indicating that countries with higher investment have significantly lower rates (31, 32). This underscores the need to strengthen public health policies and consider the social determinants of TB when allocating resources (33).

In this context, the present study carried out an analysis of TB burden from an intersectionality perspective, addressing social determinants that affect TB mortality rates in rural parishes and canton capitals in Ecuador (where canton capitals aggregate urban parishes). The high TB mortality rate in rural areas suggests that these regions face a greater risk compared to urban areas. This pattern may be influenced by multiple factors characteristic of rural areas in Ecuador, such as difficulty in accessing healthcare services, delayed diagnoses, high poverty levels, and various socio-environmental conditions that hinder timely disease detection and treatment (16, 34, 35). Additionally, the general population could be affected by intrinsic social factors such as gender, age, ethnicity, economic status, and educational level, which condition and limit the decision to seek medical care among those who may be affected by TB. Furthermore, economic growth and urbanization in recent decades have deepened the inequality between urban and rural areas, widening the gap in terms of development and access to services (6, 8–10, 35).

In Ecuador, TB has historically been associated with poverty and social vulnerability, contributing to low coverage of preventive treatment and an increase in the number of deaths (36). This study identified a direct relationship between high poverty levels and higher TB mortality. As poverty decreases, TB-related deaths also decline progressively. These findings strongly reinforce the close link between poverty and TB mortality, highlighting the need to focus public health strategies on higher poverty quintiles. Improving socioeconomic conditions in these poor parishes could significantly reduce TB-related deaths, as clearly outlined in the guidelines and plans established by WHO and PAHO (37–41).

On the other hand, our analysis reveals that TB mortality rates are higher in low population density groups. This finding challenges the assumption that population density is a key factor in disease transmission. A possible explanation lies in healthcare access, as high-density areas generally have better medical services, enabling timely diagnosis and treatment, which helps reduce mortality. In urban settings, TB may be diagnosed more frequently and treated effectively, contributing to lower mortality rates. Sociodemographic factors also play a role, as low-density areas tend to have higher poverty levels and poorer living conditions, increasing the risk of TB-related deaths. In conclusion, this relationship further reinforces the rural-urban division, as low-density areas predominantly correspond to rural parishes (35, 42–44).

In the univariate analysis, a positive relationship was observed between overcrowding and the tuberculosis mortality ratio. That is, as the percentage of homes in overcrowded conditions increases, so does the TB mortality rate. This finding suggests that the living of several people in close quarters may favor disease transmission, generating a higher risk of fatal outcomes. In other contexts, for instance, overcrowding is a common issue in most prisons across Latin America or in poor households in general, including Ecuador, and represents a risk factor for the spread of respiratory diseases such as TB. This situation becomes even more critical when there are gaps in timely diagnosis, case and contact monitoring, treatment provision, and the availability of adequate isolation spaces. Studies have estimated that the risk of developing latent TB infection is nearly three times higher among inmates living in spaces smaller than 18 square meters (45–47). If we extend these examples to limited residential spaces, as frequently seen in rural areas where families of six to eight members commonly share a single household, overcrowding becomes a critical social determinant that warrants detailed analysis (13, 30, 34, 35, 42, 43, 48).

Since parishes represent the smallest administrative unit within Ecuador's political structure (18, 20, 21, 48–51), data analysis at this level allows for a more precise and detailed evaluation of disparities in TB incidence. Unlike provincial or cantonal studies, which may mask internal differences, a parish-level approach facilitates the identification of specific areas with higher disease incidence and provides a deeper understanding of the social and economic factors influencing its distribution (35, 48, 52). Moreover, social determinants of health (such as poverty, access to healthcare services, and housing conditions) vary significantly even within the same canton or province (39, 40, 53, 54). This approach facilitates understanding the dynamics of the disease, identifying associated factors, and detecting active transmission clusters, which can strengthen disease control strategies. Although at the urban level, the INEC databases did not show individual parishes as in the case of rural parishes, we obtain an approximate view of the phenomenon we are interested in studying at this geographic level, by normalizing it with the population.

In the multivariate analysis, which included as explanatory variables poverty by UBN, territorial division (rural vs. urban), population density, and overcrowding, it was observed that three of these variables maintained a statistically significant association with tuberculosis mortality, while overcrowding did not show significance after adjustment for the other variables. The association was clear in the univariate analysis for overcrowding. However, when the other variables (poverty, population density, and geographic area) were incorporated into the multivariate model, the effect of overcrowding lost statistical significance. This indicates that its impact on TB mortality could be partially explained or mediated by other social determinants, such as structural poverty or urban-rural differences. Overall, the univariate analysis reinforces the importance of overcrowding as a relevant risk factor, although its influence appears to depend on the socioeconomic and demographic context.

Our study has some limitations that we wish to acknowledge. First, the TB mortality rate was used as the primary indicator, as it is the only publicly available information on the TB burden at the parish level. However, the TB incidence rate would have been a more appropriate metric, as it would allow for better identification of disease distribution patterns about sociodemographic variables. However, incidence data at the parish level are not accessible in Ecuador. Second, mortality data are aggregated at the cantonal capital level, without disaggregation for individual urban parishes. While the analysis allows for consistency in comparisons between rural and urban areas at the parish level, this aggregation can obscure heterogeneity within large cities such as Quito and Guayaquil, where socioeconomic conditions can vary considerably between areas.

An additional consideration is the potential influence of small population sizes on the mortality rates observed in rural areas. In our dataset, 31 rural zones had fewer than 10,000 inhabitants and reported only one death attributed to TB. Although the mortality rates were standardized per 100,000 population, which generally allows comparisons across areas with different population sizes, the small denominators in these cases can amplify random variation and generate artifactual fluctuations in the calculated rates. In fact, these rural zones showed mortality rates ranging between 10.51 and 21.4 per 100,000, despite representing only single events. To minimize the risk of distortion from extreme values, we excluded 16 zones with implausibly high rates (up to 150 per 100,000) from the analysis. Nevertheless, it is important to acknowledge that residual artifacts in rural areas may persist, and these should be interpreted with caution when drawing conclusions about spatial patterns of TB mortality.

Finally, this study focused on data analysis from 2010, as this is the most recent period for which mortality records by parish of residence are available, along with complete socioeconomic indicators at the same territorial level and year. Although the most recent 2022 Population and Housing Census offers updated socioeconomic data at the parish level, TB mortality information for that year is only available by parish of death, not by parish of residence, which biases the territorial analysis of the disease, mainly in rural areas with less access to health services, where patients may die outside their parish of residence. Alternatively, to improve the analysis using more recent data, one could consider working at the provincial level (data are available by province of residence) or using information by parish of death. However, this would entail a significant loss of spatial resolution or introduce biases based on location, which could limit the ability to identify fine-grained and coherent spatial patterns. This limitation highlights the need for institutions that manage health data to improve mechanisms for transferring and accessing these data for research purposes. In the future, if this information becomes available, the methodological framework used in this study could serve as a reference for updated analyses using an intersectionality approach.

In conclusion, this pioneering study analyzes TB mortality from an intersectional perspective, showing how often underestimated factors combine and reinforce each other. In Ecuador, it reveals an unequal impact on rural communities with low population density and high poverty, highlighting that TB should be understood primarily as a social disease with a medical dimension. These findings underscore the multifactorial nature of TB mortality and reinforce the need to address both socioeconomic and geographic inequities in public health strategies. For TB control and prevention efforts to be effective and sustainable, it is essential to integrate social determinants of health into program design. Tailored policies that prioritize underserved rural populations and structurally disadvantaged communities may be critical for reducing TB-related mortality in Ecuador and similar settings.

## Data Availability

The original contributions presented in the study are included in the article/Supplementary material, further inquiries can be directed to the corresponding author.
